# Predictors of treatment failure among patients with gunshot wounds and post-traumatic stress disorder

**DOI:** 10.1186/s12871-021-01482-8

**Published:** 2021-10-30

**Authors:** Iurii Leonidovych Kuchyn, Vasyl’ Romanovych Horoshko

**Affiliations:** National Medical University named after O.O.Bogomolets, Taras Shevchenko Boulevard, 13, Kyiv, 01601 Ukraine

## Abstract

**Background:**

The 82.1% treatment failure of post-traumatic stress disorder (PTSD), associated with gunshot wounds, is related to high incidence of chronic pain syndrome as well as resistance to the PTSD treatment. Defining treatment failure predictors among the PTSD patients with gunshot extremity wounds and the following therapy would improve treatment outcomes.

**Methods:**

A total of 218 patients completed the study. The Mississippi Scale for Combat-Related PTSD (M-PTSD) was used for assessment of the treatment outcome rate. The risk relation between treatment failure and factors was assessed by a univariate or multivariate logistic regression method, with the model accuracy measured by the AUC – Area under the ROC curve. The odds ratio (OR) was considered for the qualitative factor assessment.

**Results:**

The predictors of the PTSD treatment failure among the patients with gunshot wounds to the extremities are: 1) anesthesia type: the risk of failure is higher with the general anesthesia compared to the regional (*p* = 0.002), OR = 0.30 (95% CI 0.13-0.69) and the regional one with sedation (*p* = 0.004), OR = 0.30 (95% CI 0,14-0.65); 2) severe postoperative pain: the risk of treatment failure rises with increased pain intensity assessed by the visual analogue scale (*p* = 0.02), OR = 3.2 (95% CI 1.2-8.3).

**Conclusions:**

The analysis showed that administration of general anesthesia compared to the regional one (regardless of the sedation) and high postoperative pain intensity are associated with higher risk of the PTSD treatment failure among patients with gunshot wounds to the extremities. The preference of regional anesthesia and postoperative pain control may potentially improve the treatment outcomes.

**Trial registration:**

ClinicalTrials.gov: Retrospectively registered on December 30, 2020, NCT04689022.

**Supplementary Information:**

The online version contains supplementary material available at 10.1186/s12871-021-01482-8.

## Background

War is a strong psycho-social factor affecting all society layers [11], and, first of all, military combatants [9, 12]. The crisis, which they have experienced, predisposes for the PTSD development [1, 4, 7]. According to the WHO, 16.2% of the world population suffer from the war consequences, and the relatives of 12.5% were wounded in action [3]. The PTSD directly causes mental disadaptation of 80% of the wounded [6], later leading to the self-destructing behavior, alcohol and drug abuse as well as the other consequences [2, 8, 10]. A wide range of psychotherapy methods for the PTSD therapy has been described, though their effectiveness is questionable [5, 14, 15]. Traumas and somatic disorders of the PTSD patients accumulate their negative effect [16].

Gunshot wounds make up 54-70% of all combat injuries. According to the Armed Forces of Ukraine Medical Command, the gunshot wounds are represented as follows: 64% of extremity injuries are represented with 35.7% of the upper and 64.3% of the lower ones. Among them, 74.8% are the soft tissue injuries, 25.2% – gunshot fractures. The bone defects are noted in 11.6% of the patients, and 35-40% of the patients need subsequent reconstructive interventions.

Regardless of the PTSD treatment progress, psychotherapy and prevention of mental disorders within the psycho-social rehabilitation of the wounded patients haven’t been studied properly [13, 16].

The subjective feelings and emotional experience of the patients, caused by a combat wound always lead to the PTSD development. So, regarding the PTSD diagnosis, such patients require certain anesthesia. As 82.1% of all PTSD cases haven’t been characterized by the positive treatment outcomes, the study may be significant for the treatment of these patients.

## Methods

The anesthesia used during operations on extremities is the general or regional one. In the study a part of patients who received regional anesthesia were sedated as well, which could also affect the study results. According to anesthesia provision, the patients were divided into 3 groups. Group І received general anesthesia (*n* = 53), the sedation with constant rate infusion of 1% propofol, 1-4 mg/kg/h, guided by Bispectral analysis (60-70 – for regional anesthesia and 40-60 – for the general one). 0.005% fentanyl analgesia was injected, 3-10 mkg/kg or 0.05-0.2 mkg/kg/min during induction; and 2-10 mkg/kg/h for maintaining analgesia, by periodic bolus injection 25-100 mkg or by permanent infusion. Group II received regional anesthesia: peripheral block was performed (*n* = 73). Group III received regional anesthesia with sedation (*n* = 92). The regional anesthesia was guided by ultrasound (apparatus Mindray DP-30 with linear array probe 5-10 MHz). A needle was inserted near the nerve roots and 20-30 ml of 0.5% bupivacaine was injected. The postoperative pain management of the I group patients was provided according to the local clinical protocol: paracetamol+/−non-steroid anti-inflammatory drugs +/−opioids; of the II and III group patients –repeated peripheral block or prolonged regional anesthesia with 0.25% bupivacaine solution.

The PTSD progress and treatment effectiveness were estimated using the Mississippi Scale for Combat-Related PTSD (M-PTSD), anesthesia risks – the American Society of Anesthesiologists (ASA) classification, pain intensity – the visual analogue scale (VAS), neuropathic pain component – the Douleur Neuropathique 4 questions (DN4).

Trial registration – ClinicalTrials.gov: Retrospectively registered on December 30, 2020, NCT04689022.

### Data collection and extraction

The study was held within the bioethics expertise protocol No.125 of October 21, 2019 issued by the Commission on Biotic Expertise and Research Ethics of O.Bogomolets National medical university, Ministry of Health of Ukraine. All study data are recorded in the patients’ reports, stored in the archive of the National military medical clinical center “Main military clinical hospital”, 18 Hospital street, Kyiv, Ukraine. Statistical analysis was performed using the EZR v.1.35 software (R statistical software version 3.4.3, R Foundation for Statistical Computing, Vienna, Austria).

### Statistical analysis

Statistical analysis was performed using the EZR v.1.35 software (R statistical software version 3.4.3, R Foundation for Statistical Computing, Vienna, Austria).

A univariate or multivariate logistic regression method was used to assess the risk relation between treatment failure and factors. The model accuracy was measured by the AUC – Area under the ROC curve, with the 95% confidence interval (CI). The Odds Ratio (OR) and its 95% CI were calculated for the qualitative factor effect assessment (the significance level of 5%), *p* = 0.05.

## Results

The study is based on the authors’ clinical experience of treatment of 218 combatants with gunshot wounds to the extremities, accompanied with the PTSD, during 2014-2019, the patients operated under anesthesia.

The treatment outcome rate was assessed by the Mississippi Scale for Combat-Related PTSD (M-PTSD). A positive outcome rate is represented with the patient post-discharge positive coping, which corresponds to 94-58 points, observed in 39 patients (17.9%). A treatment failure is regarded as the absent PTSD treatment effect after discharge, which corresponds to 148-113 points, observed in 5 patients (2.3%) and psychic disorders, which correspond to 112-95 points, observed in 174 patients (79,8%). The results evidence about the 82.1% post-discharge treatment failure.

The variables of the PTSD patients with the extremity gunshot wounds, operated under anesthesia, were equal by Kruskal-Wallis test (see Table 1).

**Table 1:** Variables of patients with the PTSD, associated with the extremity gunshot wounds $\overline{X}$ ±SD

Variable	Anesthesia type			*p*
	General anesthesia (*n* = 53)	Regional anesthesia (*n* = 73)	Regional anesthesia and sedation (*n* = 92)	
Age (years)	31.7 ± 8.8	32.6 ± 10.1	33.3 ± 8.5	0.424
Height (cm)	178.2 ± 7.3	178 ± 5.6	179.9 ± 4.9	0.101
Weight (kg)	79.9 ± 10.4	80.7 ± 8.4	80.7 ± 6.3	0.414
Anesthesia duration (min)	140.7 ± 80.5	147.7 ± 75.4	145.4 ± 66.1	0.762
Operation duration (min)	121.4 ± 74.5	132.7 ± 77.2	130.4 ± 68.5	0.601

The following 17 characteristics of the PTSD treatment failure were assessed: anesthesia type, patient age, height and weight; BMI; ASA score; operation duration; anesthesia duration; systolic and diastolic arterial pressure; heart rate; pre- and post-operative pain intensity measured by the VAS scale; pre-operative neuropathic pain by the DN4, pre- and post-operative M-PTSD, pre-and post-operative blood glucose level. The results are offered in Table 2.

**Table 2:** Coefficients of univariate logistic regressions of the treatment failure risks prognosis

Factor variable		Coefficient, b ± m	*P*	OR (95% CI)
Anesthesia types	General anesthesia	Reference		
	Regional anesthesia	− 1.20 ± 0.42	0.004	0.30 (0.13-0.69)
	Regional anesthesia and sedation	−1.21 ± 0.40	0.002	0.30 (0.14-0.65)
Age		0.016 ± 0.018	0.360	–
Height		−0.009 ± 0.005	0.076	–
Weight		−0.027 ± 0.021	0.203	–
ІМТ		0.0005 ± 0.0004	0.160	–
ASA		0.67 ± 0.36	0.063	–
Anesthesia duration		0.0034 ± 0.0021	0.098	–
Surgery duration		0.0032 ± 0.0021	0.125	–
Sys АТ		−0.010 ± 0.019	0.589	–
Dia АТ		0.003 ± 0.023	0.907	–
Heart rate		0.016 ± 0.018	0.369	–
Pre-operative VAS		−0.03 ± 0.25	0.889	–
Pre-operativeDN4		0.19 ± 0.25	0.445	–
Pre-operative M-PTSD		−0.03 ± 0.26	0.919	–
Post-operative VAS		0.30 ± 0.22	0.177	–
Post-operative m-PTSD		−0.013 ± 0.028	0.634	–
Pre-operative blood glucose level		−0.12 ± 0.21	0.573	–
Post-operative blood glucose level		−0.29 ± 0.29	0.321	–

The dependent variable was represented with the M-PTSD data. If the post-treatment M-PTSD exceeded 75points, the treatment was considered to fail (dependent variable Y = 1, with 48 combatants altogether). If the post-treatment M-PTSD didn’t exceed 75 points, the treatment was considered productive (dependent variable Y = 0, with 170 combatants altogether). The authors used a univariate and multivariate logistic regression methods.

The univariate analysis revealed relationship (*p* < 0.05) with the anesthesia type. As for the combatants who were given general anesthesia, the risk is higher, compared to the regional anesthesia (*p* = 0.002), OR = 0.30 (95% CI 0.13-0.69) and regional anesthesia with sedation (*p* = 0.004), OR = 0.30 (95% CI 0.14-0.65).

**Fig. 1:**
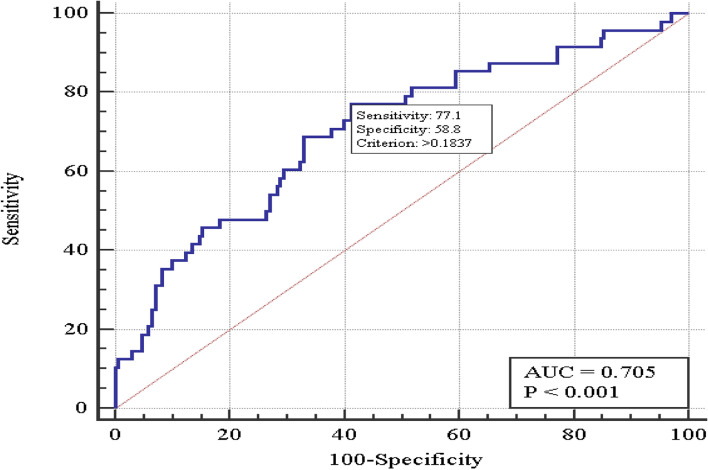
The M-PTSD treatment failure model curve (the PTSD patients with gunshot wounds to extremities)

The multivariate logistic regression was chosen for the significant variables selection (by stepwise method, with the reporting threshold of entry *p* < 0.2 and exit *p* > 0.3). Six factors have been selected: anesthesia type, height, BMI, ASA risk, heart rate, and the pre-operative M-PTSD. As a result, multi-variate valid logistic regression of treatment failure prognosis with 6 variables was built (χ^2^ = 26,7 with *p* = 0.002). Figure 1 shows the operation characteristic curve. The Area under the operation characteristic curve, AUC = 0.71 (95% CI 0.64-0.77) evidences about the relationship between the factors and treatment failure.

Table 3 shows the critical threshold as well as the model sensitivity and specificity within the threshold.

**Table 3:** Coefficients of the multi-variate six-factor logistic regression model of the PTSD treatment failure prognosis (in combatants with the gunshot wounds to extremities)

Variable		Model coefficient, b ± m	Significance level	OR (95% CI)
Anesthesia types	General anesthesia	Reference		
	Regional anesthesia	−1.50 ± 0.49	**0.002**	**0.23 (0.08-0.59)**
	Regional anesthesia and sedation	−1.46 ± 0.49	**0.003**	**0.23 (0.09-0.61)**
Height		−0.056 ± 0.030	0.059	–
BMI		0.0036 ± 0.0020	0.083	–
ASA		0.54 ± 0.38	0.155	–
HR		0.031 ± 0.020	0.120	–
Pre-operative M-PTSD		0.057 ± 0.35	0.100	–

As the table shows, the probability (standardized by 5 risk factors) of the M-PTSD treatment failure for the military combatants operated under general anesthesia is higher (*p* = 0.002), OR = 0.23 (95% CI 0.08-0.59), compared to regional anesthesia and regional anesthesia with sedation (*p* = 0.003), OR = 0.23 (95% CI 0.09-0.61).

**Fig. 2:**
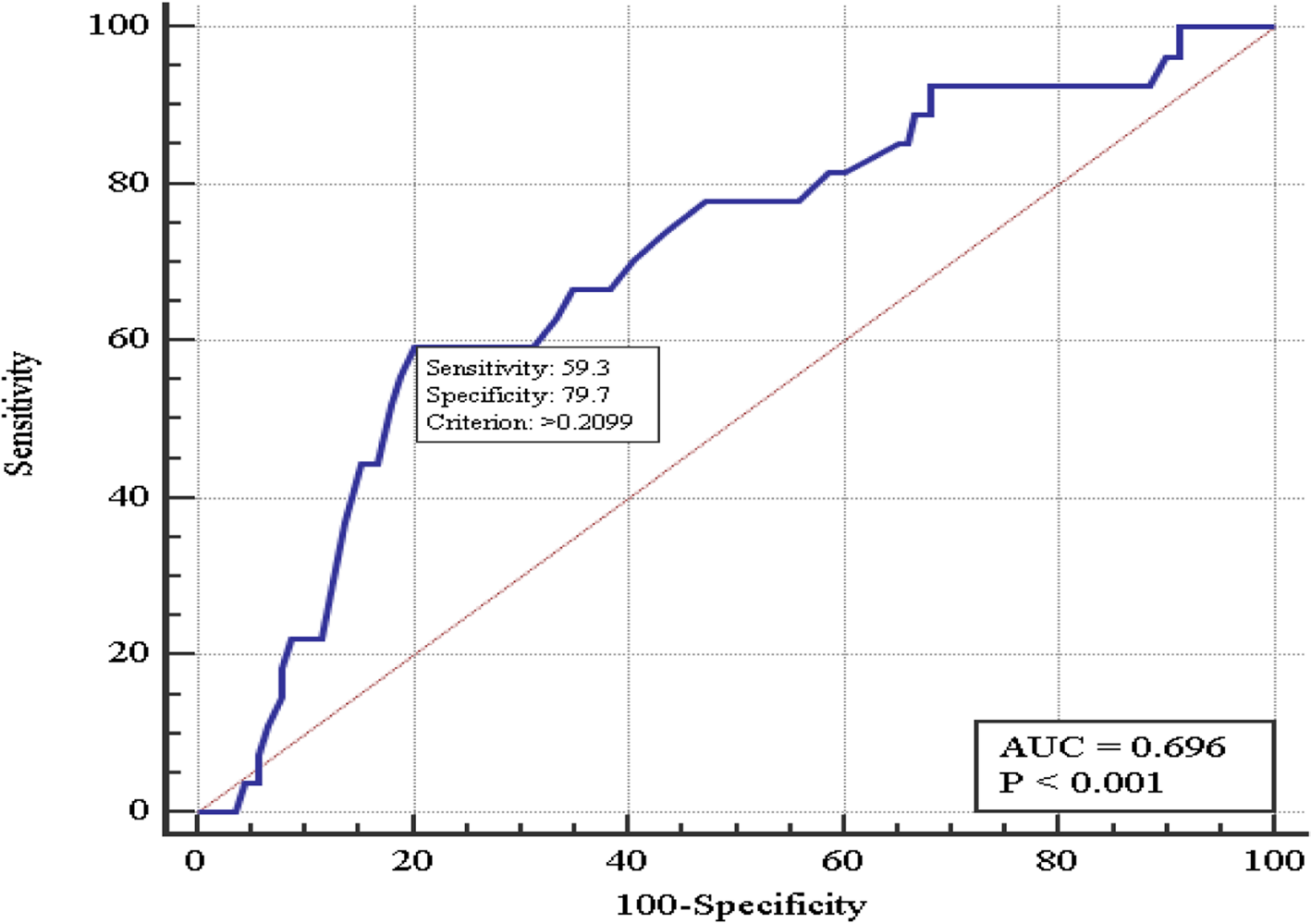
The PTSD treatment failure prognosis model curve (the patients with gunshot wounds to extremities, operated under regional anesthesia, with or without sedation)

The M-PTSD treatment outcomes after general anesthesia are significantly worse, so, it was decided to analyze treatment failure risk in the group in which regional and regional anesthesia with sedation were used (165 patients). The same risk factors were analyzed. Two factors were emphasized: the postoperative VAS-assessed pain intensity and age. A bi-variate valid logistic regression model was used (χ^2^ = 9.5, *p* = 0.009). Figure 2 shows the treatment failure bi-variate model characteristics curve. The area under the operating characteristics curve AUC = 0.70 (95% CI 0.62-0.77), which confirms relationship between the treatment failure and the factors.

Table 4 shows the critical threshold, sensitivity and specificity of the model with the chosen threshold as well as the multi-variate analysis of the treatment failure prognosis.

**Table 4:** Coefficients of the bi-variate logistic regression model of treatment failure prognosis for the PTSD combatants with the gunshot wounds to extremities, operated under regional) anesthesia (with or without sedation)

Variable	Model coefficient, b ± m	Significance level	OR (95% CI)
Age	0.044 ± 0.022	0.05	1.04 (1.00-1.09)
Post-operative VAS	1.16 ± 0.49	0.02	3.2 (1.2-8.3)

The probability of the PTSD treatment failure for the wounded combatants operated under regional anesthesia (with or without sedation) increases (*p* = 0.05) with the VAS-assessed post-operative pain intensity increase (*p* = 0.02), OR = 3.2 (95% CI 1.2-8.3) for each point (standardized by age) as well as with the age, OR = 1.04 (95% CI 1.00-1.09) for each year (standardized by the post-operative VAS-assessed pain intensity).

## Discussion

It has been noted that the PTSD in military combatants results from their direct participation in military actions [1, 4, 7]. The study revealed that if a military combatant was wounded during the action operation, his emotional and subjective feelings will 100% lead to the PTSD. Other scientists [2, 8, 10] state about the PTSD remote treatment failure in 80% of the military combatants. The study evidences about 82.1% of the PTSD m-related wounds treatment failure, which was essential for the subsequent studies. Some authors [5, 14, 15] state about treatment effectiveness of psychopharmacological drugs and psychotherapy, which is rather questionable. Traumas and somatic diseases in the PTSD patients are known to accumulate their negative effects [16]. So, definition of the PTSD treatment failure predictors may improve treatment outcomes.

The following 17 characteristics of the PTSD treatment failure were assessed for defining the PTSD treatment failure predictors: anesthesia type, patient age, height and weight; BMI; ASA score; operation duration; anesthesia duration; systolic and diastolic arterial pressure; heart rate; pre- and post-operative pain intensity by the VAS scale; pre-operative neuropathic pain by theDN4, pre- and post-operative M-PTSD, pre-and post-operative blood glucose level. The study revealed that the probability of the PTSD treatment failure is higher for the military combatants operated under general anesthesia (standardized by 5 risk factors) compared to regional anesthesia (*p* = 0.002) OR = 0.23 (95% CI 0.08-0.59) and regional anesthesia with sedation (*p* = 0.003), OR = 0.23 (95% CI 0.09-0.61). The probability of the PTSD treatment failure for the wounded combatants operated under regional anesthesia (with or without sedation) increases (*p* = 0.05) with the VAS –assessed post-operative pain intensity increase (*p* = 0.02), OR = 3.2 (95% CI 1.2-8.3) for each point (standardized by age).

So, the use of general anesthesia compared to regional (regardless of sedation) and high postoperative pain intensity are associated with a higher risk of the PTSD treatment failure in patients with the gunshot wounds to extremities. The study evidences that the choice of regional anesthesia and post-operative pain control may significantly improve treatment outcomes in such patients.

## Conclusions

The analysis of 218 PTSD patients with gunshot wounds, operated under anesthesia, showed that the use of general anesthesia compared to regional (regardless of sedation) and high postoperative pain intensity are associated with a higher risk of the PTSD treatment failure in patients with the gunshot wounds to extremities. The choice of regional anesthesia and post-operative pain control may significantly improve treatment outcomes in such patients.

## Supplementary Information

**Additional file 1.**

## Data Availability

The datasets used and analysed during the current study are available from the corresponding author on reasonable request.
