# Supplementary material for: Predictors of treatment failure among patients with gunshot wounds and post-traumatic stress disorder
Source: BMC Anesthesiol. 2021 Oct 30;21:263. doi: 10.1186/s12871-021-01482-8 (PMC8557033; doi:10.1186/s12871-021-01482-8)
Supplement: Supplementary file 1 — Additional file 1. [file 12871_2021_1482_MOESM1_ESM.docx]

**Research protocol**

**Research protocol: part 1**

**Project summary**

Background**:** The 82.1% treatment failure of post-traumatic stress disorder(PTSD), associated with gunshot wounds, is related to high incidence of chronic pain syndrome as well as resistance to the PTSD treatment. Defining treatment failure predictors among the PTSD patients with gunshot extremity wounds and the following therapy would improve treatment outcomes.

Methods: A total of 218 patients completed the study. The Mississippi Scale for Combat-Related PTSD (M-PTSD) was used for assessment of the treatment outcome rate. The risk relation between treatment failure and factors was assessed by a univariate or multivariate logistic regression method, with the model accuracy measured by the AUC – Area under the ROC curve. The odds ratio (OR) was considered for the qualitative factor assessment.

Results: The predictors of the PTSD treatment failure among the patients with gunshot wounds to the extremities are: 1) anesthesia type: the risk of failure is higher with the general anesthesia compared to the regional (p=0.002), OR=0.30 (95% CI 0.13-0.69) and the regional one with sedation (p=0.004), OR=0.30 (95% CI 0,14-0.65); 2) severe postoperative pain: the risk of treatment failure rises with increased pain intensity assessed by the visual analogue scale (p=0.02), OR=3.2 (95% CI 1.2-8.3).

Conclusions: The analysis showed that administration of general anesthesia compared to the regional one (regardless of the sedation) and high postoperative pain intensity are associated with higher risk of the PTSD treatment failure among patients with gunshot wounds to the extremities. The preference of regional anesthesia and postoperative pain control may potentially improve the treatment outcomes.

**General information**

Protocol title: Predictors of treatment failure among patients with gunshot wounds and post-traumatic stress disorder

Protocol identifying number (if any), and date: ClinicalTrials.gov: Retrospectively registered on December 30, 2020, NCT04689022

Name and address of the sponsor/funder: Vasyl’ Horoshko

Name and title of the investigator(s) who is (are) responsible for conducting the research: Vasyl’ Horoshko – collection and processing of the data, analysis, composing the paper. Iurii Kuchyn – study concept and design.

Address and telephone number(s) of the research site(s): National military medical clinical center “Main military clinical hospital”, 18 Hospital street, Kyiv, Ukraine. Tel.+380972615452. Correspondence to e-mail: [dr.horoshko@ukr.net](mailto:dr.horoshko@ukr.net)

**Rationale & background information**

War is a strong psycho-social factor affecting all society layers [11], and, first of all, military combatants [9, 12]. The crisis, which they have experienced, predisposes for the PTSD development [1, 4, 7]. According to the WHO, 16.2 % of the world population suffer from the war consequences, and the relatives of 12.5 % were wounded in action [3]. The PTSD directly causes mental disadaptation of 80% of the wounded [6], later leading to the self-destructing behavior, alcohol and drug abuse as well as the other consequences [2, 8, 10]. A wide range of psychotherapy methods for the PTSD therapy has been described, though their effectiveness is questionable [5, 14, 15]. Traumas and somatic disorders of the PTSD patients accumulate their negative effect [16].

Gunshot wounds make up 54-70% of all combat injuries. According to the Armed Forces of Ukraine Medical Command, the gunshot wounds are represented as follows: 64% of extremity injuries are represented with 35.7% of the upper and 64.3% of the lower ones. Among them, 74.8% are the soft tissue injuries, 25.2% – gunshot fractures. The bone defects are noted in 11.6% of the patients, and 35-40% of the patients need subsequent reconstructive interventions.

Regardless of the PTSD treatment progress, psychotherapy and prevention of mental disorders within the psycho-social rehabilitation of the wounded patients haven’t been studied properly [13, 16].

The subjective feelings and emotional experience of the patients, caused by a combat wound always lead to the PTSD development. So, regarding the PTSD diagnosis, such patients require certain anesthesia. As 82.1% of all PTSD cases haven’t been characterized by the positive treatment outcomes, the study may be significant for the treatment of these patients.

**Study goals and objectives**

Identify predictors of treatment failure among patients with gunshot wounds and post-traumatic stress disorder

**Study design**

The study is based on the authors’ clinical experience of treatment of 218 combatants with gunshot wounds to the extremities, accompanied with the PTSD, during 2014-2019, the patients operated under anesthesia.

The treatment outcome rate was assessed by the Mississippi Scale for Combat-Related PTSD (M-PTSD). A positive outcome rate is represented with the patient post-discharge positive coping, which corresponds to 94-58 points, observed in 39 patients (17.9%). A treatment failure is regarded as the absent PTSD treatment effect after discharge, which corresponds to 148-113 points, observed in 5 patients (2.3%) and psychic disorders, which correspond to 112-95 points, observed in 174 patients (79,8%). The results evidence about the 82.1% post-discharge treatment failure.

The study was held within the bioethics expertise protocol No.125 of October 21, 2019 issued by the Commission on Biotic Expertise and Research Ethics of O.Bogomolets National medical university, Ministry of Health of Ukraine. All study data are recorded in the patients’ reports, stored in the archive of the National military medical clinical center “Main military clinical hospital”, 18 Hospital street, Kyiv, Ukraine. Statistical analysis was performed using the EZR v.1.35 software (R statistical software version 3.4.3, R Foundation for Statistical Computing, Vienna, Austria). Statistical analysis was performed using the EZR v.1.35 software (R statistical software version 3.4.3, R Foundation for Statistical Computing, Vienna, Austria). A univariate or multivariate logistic regression method was used to assess the risk relation between treatment failure and factors. The model accuracy was measured by the AUC – Area under the ROC curve, with the 95% confidence interval (CI). The Odds Ratio (OR) and its 95% CI were calculated for the qualitative factor effect assessment( the significance level of 5%), p=0.05.

**Methodology**

The anesthesia used during operations on extremities is the general or regional one. In the study a part of patients who received regional anesthesia were sedated as well, which could also affect the study results. According to anesthesia provision, the patients were divided into 3 groups. Group І received general anesthesia (n=53), the sedation with constant rate infusion of 1% propofol, 1-4mg/kg/h, guided by Bispectral analysis **(60-70 – for regional anesthesia and 40-60 – for the general one). 0.005% fentanyl analgesia was injected, 3-10 mkg/kg or 0.05-0.2 mkg/kg/min during induction; and 2-10 mkg/kg/h for maintaining analgesia, by periodic bolus injection 25-100 mkg or by permanent infusion. Group II received regional anesthesia: peripheral block was performed** (n=73). Group III received regional anesthesia with sedation (n=92). The regional anesthesia was guided by ultrasound (apparatus Mindray DP-30 with linear array probe 5-10 MHz). A needle was inserted near the nerve roots and 20-30 ml of 0.5% bupivacaine was injected. The postoperative pain management of the I group patients was provided according to the local clinical protocol: paracetamol+/-non-steroid anti-inflammatory drugs +/-opioids; of the II and III group patients –repeated peripheral block or prolonged regional anesthesia with 0.25% bupivacaine solution.

The PTSD progress and treatment effectiveness were estimated using the Mississippi Scale for Combat-Related PTSD (M-PTSD), anesthesia risks – the American Society of Anesthesiologists (ASA) classification, pain intensity – the visual analogue scale (VAS), neuropathic pain component – the Douleur Neuropathique 4 questions (DN4).

Trial registration – ClinicalTrials.gov: Retrospectively registered on December 30, 2020, NCT04689022

The treatment outcome rate was assessed by the Mississippi Scale for Combat-Related PTSD (M-PTSD). A positive outcome rate is represented with the patient post-discharge positive coping, which corresponds to 94-58 points, observed in 39 patients (17.9%). A treatment failure is regarded as the absent PTSD treatment effect after discharge, which corresponds to 148-113 points, observed in 5 patients (2.3%) and psychic disorders, which correspond to 112-95 points, observed in 174 patients (79,8%). The results evidence about the 82.1% post-discharge treatment failure.

The variables of the PTSD patients with the extremity gunshot wounds, operated under anesthesia, were equal by Kruskal-Wallis test (see table 1).

Table 1.

Variables of patients with the PTSD, associated with the extremity gunshot wounds

$\overline{X}$±SD

| Variable | Anesthesia type | | | p |
| --- | --- | --- | --- | --- |
|  | General anesthesia  (n=53) | Regional anesthesia  (n=73) | Regional anesthesia and sedation  (n=92) |  |
| Age (years) | 31.7±8.8 | 32.6±10.1 | 33.3±8.5 | 0.424 |
| Height (cm) | 178.2±7.3 | 178±5.6 | 179.9±4.9 | 0.101 |
| Weight (kg) | 79.9±10.4 | 80.7±8.4 | 80.7±6.3 | 0.414 |
| Anesthesia duration (min) | 140.7±80.5 | 147.7±75.4 | 145.4±66.1 | 0.762 |
| Operation duration (min) | 121.4±74.5 | 132.7±77.2 | 130.4±68.5 | 0.601 |

The following 17 characteristics of the PTSD treatment failure were assessed: anesthesia type, patient age, height and weight; BMI; ASA score; operation duration; anesthesia duration; systolic and diastolic arterial pressure; heart rate; pre- and post-operative pain intensity measured by the VAS scale; pre-operative neuropathic pain by the DN4, pre- and post-operative M-PTSD , pre-and post-operative blood glucose level. The results are offered in table 2.

The dependent variable was represented with the M-PTSD data. If the post-treatment M-PTSD exceeded 75points, the treatment was considered to fail (dependent variable Y=1, with 48 combatants altogether). If the post-treatment M-PTSD didn’t exceed 75 points, the treatment was considered productive (dependent variable Y=0, with 170 combatants altogether). The authors used a univariate and multivariate logistic regression methods.

Table 2.

Coefficients of univariate logistic regressions of the treatment failure risks prognosis

| Factor variable | | Coefficient, b±m | P | OR  (95% CI) |
| --- | --- | --- | --- | --- |
| Anesthesia types | General anesthesia | Reference | | |
|  | Regional anesthesia | –1.20±0.42 | 0.004 | 0.30 (0.13-0.69) |
|  | Regional anesthesia and sedation | –1.21±0.40 | 0.002 | 0.30 (0.14-0.65) |
| Age | | 0.016±0.018 | 0.360 | – |
| Height | | -0.009±0.005 | 0.076 | – |
| Weight | | -0.027±0.021 | 0.203 | – |
| ІМТ | | 0.0005±0.0004 | 0.160 | – |
| ASA | | 0.67±0.36 | 0.063 | – |
| Anesthesia duration | | 0.0034±0.0021 | 0.098 | – |
| Surgery duration | | 0.0032±0.0021 | 0.125 | – |
| Sys АТ | | -0.010±0.019 | 0.589 | – |
| Dia АТ | | 0.003±0.023 | 0.907 | – |
| Heart rate | | 0.016±0.018 | 0.369 | – |
| Pre-operative VAS | | -0.03±0.25 | 0.889 | – |
| Pre-operativeDN4 | | 0.19±0.25 | 0.445 | – |
| Pre-operative M-PTSD | | -0.03±0.26 | 0.919 | – |
| Post-operative VAS | | 0.30±0.22 | 0.177 | – |
| Post-operative m-PTSD | | -0.013±0.028 | 0.634 | – |
| Pre-operative blood glucose level | | -0.12±0.21 | 0.573 | – |
| Post-operative blood glucose level | | -0.29±0.29 | 0.321 | – |

The univariate analysis revealed relationship (p<0.05) with the anesthesia type. As for the combatants who were given general anesthesia, the risk is higher, compared to the regional anesthesia (p=0.002), OR=0.30 (95% CI 0.13-0.69) and regional anesthesia with sedation (p=0.004), OR=0.30 (95% CI 0.14-0.65).

The multivariate logistic regression was chosen for the significant variables selection (by stepwise method, with the reporting threshold of entry p<0.2 and exit p>0.3). Six factors have been selected: anesthesia type, height, BMI, ASA risk, heart rate, and the pre-operative M-PTSD. As a result, multi-variate valid logistic regression of treatment failure prognosis with 6 variables was built (χ^2^=26,7 with p=0.002). Figure 1 shows the operation characteristic curve. The Area under the operation characteristic curve, AUC=0.71 (95% CI 0.64-0.77) evidences about the relationship between the factors and treatment failure.


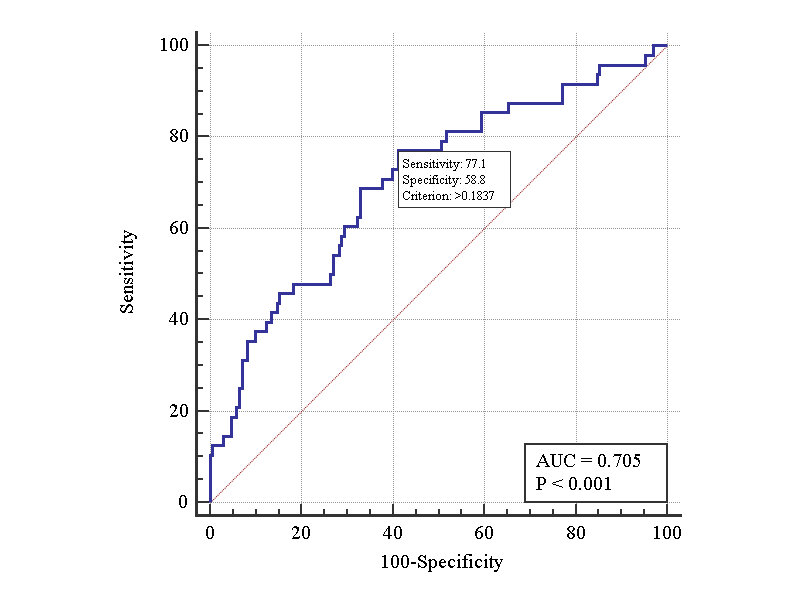


Figure 1. The M-PTSD treatment failure model curve (the PTSD patients with gunshot wounds to extremities).

Table 3 shows the critical threshold as well as the model sensitivity and specificity within the threshold.

Table 3.

Coefficients of the multi-variate six-factor logistic regression model of the PTSD treatment failure prognosis (in combatants with the gunshot wounds to extremities).

| Variable | | Model coefficient, b±m | Significance level | OR  (95% CI) |
| --- | --- | --- | --- | --- |
| Anesthesia types | General anesthesia | Reference | | |
|  | Regional anesthesia | –1.50±0.49 | **0.002** | **0.23 (0.08-0.59)** |
|  | Regional anesthesia and sedation | –1.46±0.49 | **0.003** | **0.23 (0.09-0.61)** |
| Height | | -0.056±0.030 | 0.059 | – |
| BMI | | 0.0036±0.0020 | 0.083 | – |
| ASA | | 0.54±0.38 | 0.155 | – |
| HR | | 0.031±0.020 | 0.120 | – |
| Pre-operative M-PTSD | | 0.057±0.35 | 0.100 | – |

As the table shows, the probability (standardized by 5 risk factors) of the M-PTSD treatment failure for the military combatants operated under general anesthesia is higher (p=0.002), OR=0.23 (95% CI 0.08-0.59), compared to regional anesthesia and regional anesthesia with sedation (p=0.003), OR=0.23 (95% CI 0.09-0.61).

The M-PTSD treatment outcomes after general anesthesia are significantly worse, so, it was decided to analyze treatment failure risk in the group in which regional and regional anesthesia with sedation were used (165 patients). The same risk factors were analyzed. Two factors were emphasized: the postoperative VAS-assessed pain intensity and age. A bi-variate valid logistic regression model was used (χ^2^=9.5, p=0.009). Figure 2 shows the treatment failure bi-variate model characteristics curve. The area under the operating characteristics curve AUC=0.70 (95% CI 0.62-0.77), which confirms relationship between the treatment failure and the factors.


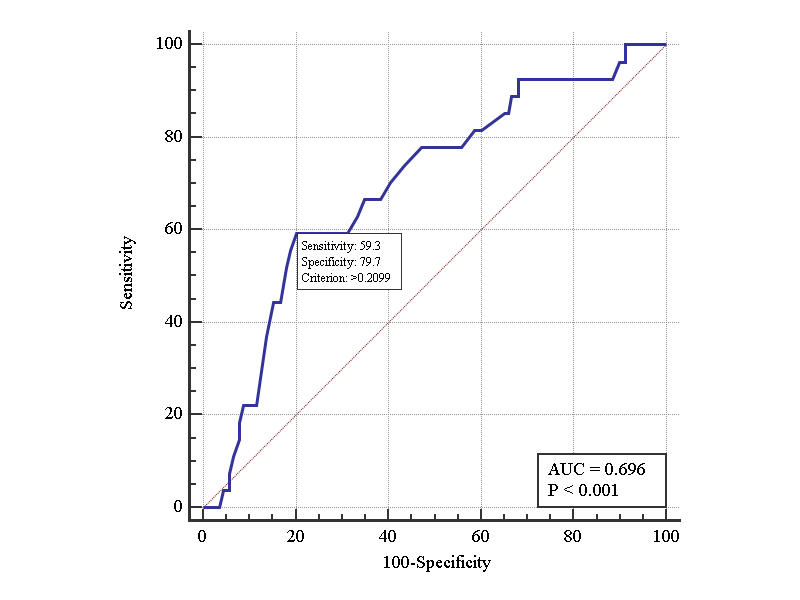
Figure 2. The PTSD treatment failure prognosis model curve (the patients with gunshot wounds to extremities, operated under regional anesthesia, with or without sedation).

Table 4 shows the critical threshold, sensitivity and specificity of the model with the chosen threshold as well as the multi-variate analysis of the treatment failure prognosis.

Table 4.

Coefficients of the bi-variate logistic regression model of treatment failure prognosis for the PTSD combatants with the gunshot wounds to extremities, operated under regional) anesthesia(with or without sedation).

| Variable | Model coefficient, b±m | Significance level | OR  (95% CI) |
| --- | --- | --- | --- |
| Age | 0.044±0.022 | 0.05 | 1.04 (1.00-1.09) |
| Post-operative VAS | 1.16±0.49 | 0.02 | 3.2 (1.2-8.3) |

The probability of the PTSD treatment failure for the wounded combatants operated under regional anesthesia(with or without sedation) increases (p=0.05) with the VAS-assessed post-operative pain intensity increase (p=0.02), OR=3.2 (95% CI 1.2-8.3) for each point( standardized by age) as well as with the age, OR=1.04 (95% CI 1.00-1.09) for each year (standardized by the post-operative VAS-assessed pain intensity).

**Safety considerations**

The safety of the participants was ensured in accordance with the local clinical protocol. The peculiarities of the research were explained to all participants. Positive responses were received.

**Follow-up**

All patients were monitored throughout the study period.

**Data management and statistical analysis**

The study was held within the bioethics expertise protocol No.125 of October 21, 2019 issued by the Commission on Biotic Expertise and Research Ethics of O.Bogomolets National medical university, Ministry of Health of Ukraine. All study data are recorded in the patients’ reports, stored in the archive of the National military medical clinical center “Main military clinical hospital”, 18 Hospital street, Kyiv, Ukraine. Statistical analysis was performed using the EZR v.1.35 software (R statistical software version 3.4.3, R Foundation for Statistical Computing, Vienna, Austria).

Statistical analysis was performed using the EZR v.1.35 software (R statistical software version 3.4.3, R Foundation for Statistical Computing, Vienna, Austria).

A univariate or multivariate logistic regression method was used to assess the risk relation between treatment failure and factors. The model accuracy was measured by the AUC – Area under the ROC curve, with the 95% confidence interval (CI). The Odds Ratio (OR) and its 95% CI were calculated for the qualitative factor effect assessment( the significance level of 5%), p=0.05.

**Expected outcomes of the study**

It has been noted that the PTSD in military combatants results from their direct participation in military actions [1, 4, 7]. The study revealed that if a military combatant was wounded during the action operation, his emotional and subjective feelings will 100% lead to the PTSD. Other scientists [2, 8, 10] state about the PTSD remote treatment failure in 80% of the military combatants. The study evidences about 82.1% of the PTSD m-related wounds treatment failure, which was essential for the subsequent studies. Some authors [5, 14, 15] state about treatment effectiveness of psychopharmacological drugs and psychotherapy, which is rather questionable. Traumas and somatic diseases in the PTSD patients are known to accumulate their negative effects [16]. So, definition of the PTSD treatment failure predictors may improve treatment outcomes.

The following 17 characteristics of the PTSD treatment failure were assessed for defining the PTSD treatment failure predictors: anesthesia type, patient age, height and weight; BMI; ASA score; operation duration; anesthesia duration; systolic and diastolic arterial pressure; heart rate; pre- and post-operative pain intensity by the VAS scale; pre-operative neuropathic pain by theDN4, pre- and post-operative M-PTSD, pre-and post-operative blood glucose level. The study revealed that the probability of the PTSD treatment failure is higher for the military combatants operated under general anesthesia (standardized by 5 risk factors) compared to regional anesthesia (p=0.002) OR=0.23 (95% CI 0.08-0.59) and regional anesthesia with sedation (p=0.003), OR=0.23 (95% CI 0.09-0.61). The probability of the PTSD treatment failure for the wounded combatants operated under regional anesthesia(with or without sedation) increases (p=0.05) with the VAS –assessed post-operative pain intensity increase (p=0.02), OR=3.2 (95% CI 1.2-8.3) for each point( standardized by age).

So, the use of general anesthesia compared to regional (regardless of sedation) and high postoperative pain intensity are associated with a higher risk of the PTSD treatment failure in patients with the gunshot wounds to extremities. The study evidences that the choice of regional anesthesia and post-operative pain control may significantly improve treatment outcomes in such patients.

**Quality assurance**

Familiarity with the international principles of clinical / experimental research (GCP). State Expert Center of the Ministry of Health of Ukraine “Good Clinical Practice of GCP. Regulatory regulation of clinical trials ”/” Good Clinical Practice (GCP). Clinical trial regulation ”. 05.12.2017. Kiev. Certificate №3298.

**Dissemination of results and publication policy**

The analysis of 218 PTSD patients with gunshot wounds, operated under anesthesia, showed that the use of general anesthesia compared to regional (regardless of sedation) and high postoperative pain intensity are associated with a higher risk of the PTSD treatment failure in patients with the gunshot wounds to extremities. The choice of regional anesthesia and post-operative pain control may significantly improve treatment outcomes in such patients.

**Duration of the project**

The study has already been completed.

**Problems anticipated**

No problems are expected.

**Ethics**

Issues that may cause ethical concern are not predicted. Conscious consent from study participants (patients) to participate in the study was obtained in all cases.

The study was held within the bioethics expertise protocol No.125 of October 21, 2019 issued by the Commission on Biotic Expertise and Research Ethics of O.Bogomolets National medical university, Ministry of Health of Ukraine.

I confirm that all methods have been performed in accordance with the relevant guidelines and standards by including a statement in the Methods section.

**References**

1. Agaibi CE, Wilson JP. Trauma, PTSD, and resilience: a review of the literature. Trauma Violence Abuse. 2005 Jul; 6(3):195-216. doi: 10.1177/1524838005277438. PMID: 16237155.
2. Back SE, Brady KT, Sonne SC, Verduin ML. Symptom improvement in co-occurring PTSD and alcohol dependence. J Nerv Ment Dis. 2006 Sep;194(9):690-6. doi: 10.1097/01.nmd.0000235794.12794.8a. Erratum in: J Nerv Ment Dis. 2006 Nov;194(11):825. PMID: 16971821.
3. Badiuk Ml, Shevchuk OS. The combat stress reaction as a scientific problem of the world, and its social and medical consequences. Clinical and Experimental Pathology .2016; 4: 10-14.
4. Barton S, Boals A, Knowles L. Thinking about trauma: the unique contributions of event centrality and posttraumatic cognitions in predicting PTSD and posttraumatic growth. J Trauma Stress. 2013 Dec; 26(6):718-26. doi: 10.1002/jts.21863. PMID: 24490252.
5. Beck AT, Emery G, Greenberg RL. Anxiety disorders and phobias: A cognitive approach. Basic, New York. 2015, 86p.
6. Dalenberg C, Carlson EB. Dissociation in posttraumatic stress disorder part 2: How theoretical models fit the empirical evidence and recommendations for modifying the diagnostic criteria for PTSD. Psychological Trauma: Theory, Research, Practice, and Policy. 2012; 4(6), 551–559. [https://doi.org/10.1037/a0027900](https://doi.apa.org/doi/10.1037/a0027900)
7. Gillihan SJ, Cahill SP, Foa EB. Psychological theories of PTSD. New York: Guilford Press, 2014: 166-185.
8. Haller M, Chassin L. The influence of PTSD symptoms on alcohol and drug problems: Internalizing and externalizing pathways. Psychological Trauma: Theory, Research, Practice, and Policy. 2013; 5(5), 484-493. <https://doi.org/10.1037/a0029335>
9. Hoge CW, Auchterlonie JL, Milliken CS. Mental health problems, use of mental health services, and attrition from military service after returning from deployment to Iraq or Afghanistan. JAMA. 2006 Mar 1;295(9):1023-32. doi: 10.1001/jama.295.9.1023. PMID: 16507803.
10. Kolyadko SP, Denisenko MM, Kalenskaya GYu, Yavdak IO Clinical and psychopathological features of patients with neurotic disorders with comorbid addictive disorders. Ukrainian Bulletin of Psychoneurology. 2016; 24 (1): 64-68.
11. Matyash MM, Khudenko LI. Social stress disorders in the structure of the Ukrainian syndrome. Ukrainian Journal. 2016; 3: p.113.
12. Minko OI, Linskyyi IV, Baranenko OV. Alcoholic behavior and its consequences in the ATO veterans and civilians affected by the "hybrid war". Ukrainian Bulletin of Psychoneurology. 2017; 25, 190p.
13. Marcia J.E. Life transitions and stress in the context of psychosocial development. In T. W. Miller (Ed.). Handbook of stressful transitions across the lifespan. Springer Science+Business Media. 2010: 19-34. [https://doi.org/10.1007/978-1-4419-0748-6_2](https://psycnet.apa.org/doi/10.1007/978-1-4419-0748-6_2)
14. Rothbaum BO, Schwartz AC. Exposure therapy for posttraumatic stress disorder. Am J Psychother. 2002; 56(1):59-75. doi: 10.1176/appi.psychotherapy.2002.56.1.59. PMID: 11977784.
15. Tеng EJ. Efficacy of posttraumatic stress disorder treatment for comorbid panic disorder: a critical review and future directions for treatment research. Clin Psycho Sci Pract., 2016: 68-84. <https://doi.org/10.1111/cpsp.12039>

Voloshyn PV, Maruta NO. The main directions of scientific research in neurology, psychiatry and narcology of Ukraine. Ukrainian Bulletin of Psychoneurology. 2017, 25 (1): 10-18.

**Research protocol: part 2**

**Budget**

Not applicable.

**Other support for the project**

Not applicable.

**Collaboration with other scientists or research institutions**

Not applicable.

**Links to other projects**

Not applicable.

**Curriculum Vitae of investigators**

1. Last name, first name, patronymic Horoshko Vasyl’ Romanovych

2. Date of birth October 8, 1984

3. Education, year of graduation, name of educational institution: higher, in 2007 he graduated from NMU named after OO Bogomolets, in 2009 - Lviv National Medical University (internship) in the specialty "Anesthesiology and Intensive Care", in 2012 year Ukrainian Military Medical Academy (master's degree) in "Anesthesiology"

4. Specialty at the place of work: anesthesiologist

5. Postgraduate medical education: in 2009 he graduated from the Lviv National Medical University (internship) with a degree in "anesthesiology and intensive care", in 2012 the Ukrainian Military Medical Academy (master's degree) with a degree in "anesthesiology"

6. Scientific degree: no

7. Place of work and position: National Military Medical Clinical Center "Main Military Clinical Hospital" of the Ministry of Defense of Ukraine. Head of the Department of Resuscitation and Intensive Care (for medical evacuation) of the clinic of emergency care, intensive care, anesthesiology, resuscitation and detoxification

8. Mob. phone and e-mail for contacts:

+38 (097) 261-54-52; e-mail – dr.horoshko@ukr.net

9. Participation in previous research in the past (indicate place and year): did not take

10. Familiarity with the international principles of clinical / experimental research (GCP). State Expert Center of the Ministry of Health of Ukraine “Good Clinical Practice of GCP. Regulatory regulation of clinical trials ”/” Good Clinical Practice (GCP). Clinical trial regulation ”. 05.12.2017. Kiev. Certificate №3298.

**Other research activities of the investigators**

Not applicable.

**Financing and insurance**

Not applicable.
